# Operational Modal Analysis of Civil Engineering Structures with Closely Spaced Modes Based on Improved Hilbert–Huang Transform

**DOI:** 10.3390/s24237600

**Published:** 2024-11-28

**Authors:** Xu-Qiang Shang, Tian-Li Huang, Yi-Bin He, Hua-Peng Chen

**Affiliations:** 1 School of Civil Engineering, Central South University, Changsha 410075, China; shangxq@csu.edu.cn (X.-Q.S.); htianli@csu.edu.cn (T.-L.H.); 2 Hunan Architectural Design Institute Group Co., Ltd., Changsha 410012, China; 3 School of Transportation Engineering, East China Jiaotong University, Nanchang 330013, China; hp.chen@outlook.com

**Keywords:** operational modal analysis, civil engineering structures, closely spaced modes, improved Hilbert–Huang transform, analytical mode decomposition (AMD)

## Abstract

In long-span bridges and high-rise buildings, closely spaced modes are commonly observed, which greatly increases the challenge of identifying modal parameters. Hilbert–Huang transform (HHT), a widely used method for modal parameter identification, first applies empirical mode decomposition (EMD) to decompose the acquired response and then uses the Hilbert transform (HT) to identify the modal parameters. However, the problem is that the deficiency of mode separation of EMD in HHT limits its application for structures with closely spaced modes. In this study, an improved HHT based on analytical mode decomposition (AMD) is proposed and is used to identify the modal parameters of structures with closely spaced modes. In the improved HHT, AMD is first employed to replace EMD for decomposing the measured response into several mono-component modes. Then, the random decrement technique is applied to the decomposed mono-component modes to obtain the free decay responses. Furthermore, the resulting free decay responses are analyzed by HT to estimate the modal parameters of structures with closely spaced modes. Examples of a simple three-degree-of-freedom system with closely spaced modes, a high-rise building under ambient excitation, and the Ting Kau bridge under typhoon excitations are adopted to validate the accuracy, effectiveness, and applicability of the proposed method. The results demonstrate that the proposed method can efficiently and accurately identify the natural frequencies and damping ratios of structures with closely spaced modes. Moreover, its identification results are more precise compared to those obtained using existing methods.

## 1. Introduction 

Vibration-based modal analysis plays a crucial role in understanding the dynamic behavior of civil engineering structures by identifying key modal parameters such as natural frequencies, damping ratios, and mode shapes [1,2,3,4]. These modal parameters are vital for dynamic response prediction, finite element model updating, structural health monitoring, and the design of structures subjected to dynamic loads such as wind, traffic, and seismic forces. The challenge of closely spaced modes, particularly in large-span bridges and tall buildings, has attracted considerable attention in recent decades [5,6]. Closely spaced modes can result in overlapping frequency peaks, which complicates the accurate identification of modal parameters. This phenomenon often occurs in flexible, large-scale structures, where multiple modes of vibration exist within a narrow frequency range. The inability to accurately distinguish between these modes can reduce the precision of the identified modal parameters, leading to errors in dynamic analysis and monitoring. 

Traditional methods of vibration-based modal analysis include experimental modal analysis and operational modal analysis (OMA). The experimental modal analysis relies on controlled excitation to identify modal parameters but is impractical for large civil engineering structures due to the need for artificial excitation [7,8]. In contrast, OMA uses ambient excitations (such as wind, traffic, or other environmental factors) to identify modal parameters, eliminating the need for external excitation sources. Over the past few decades, several OMA techniques have emerged, including the natural excitation technique [9], frequency domain decomposition [10], autoregressive moving average vector model (ARMAV) [11], the stochastic subspace identification (SSI) method, and so on [12,13]. These OMA techniques do not interfere with the normal operation of the structures and do not cause any negative effects on the structures themselves. Consequently, OMA has garnered significant attention in the fields of civil, mechanical, and aerospace engineering [14,15,16,17,18].

To simplify signal processing, traditional OMA methods like SSI and ARMAV assume that the input is white noise and the structural response is stationary [19]. However, the response of structures under ambient excitation is typically non-stationary. Therefore, the Hilbert–Huang transform (HHT), a nonlinear and non-stationary signal analysis technique, is proposed and applied to identify the modal parameters of civil engineering structures [20,21]. The HHT method involves two steps: (1) empirical mode decomposition (EMD) decomposes the measured response into several mono-component modes, and (2) the Hilbert transform (HT) is applied to these mono-component modes to identify the modal parameters. Yang et al. [22] proposed an HHT-based system identification method for linear multi-degree-of-freedom systems using the measured free vibration time histories, in which the modal parameters (natural frequencies, mode shapes, and damping ratios) and the physical parameters (mass, stiffness, and damping matrix) of the structure are obtained. Wei et al. [23] proposed an HHT-based method to improve the accuracy of modal identification in dam structures by overcoming modal confusion issues, particularly in weak, noisy vibration signals. Xu et al. [24] adopted the HHT method in conjunction with the random decrement technique to identify the dynamic characteristics of the Di Wang building, Shenzhen, China, by using the measured data under Typhoon York. Bahar and Ramezani [25] proposed an enhanced HHT method and applied it to the modal identification of a typical three-degree-of-freedom (3-DOF) structural model subjected to a random excitation and a real 15-story building. Mao et al. [26] employed the HHT method to analyze the dynamic characteristics of the Sutong cable-stayed bridge over a continuous period of one year under normal operational conditions. Ondra et al. [27] explored the identification of complex nonlinear modes from experimental free decay responses using the HHT. 

Despite the successful applications of the HHT method for modal parameter identification in civil engineering structures, there are still some issues. For example, EMD in HHT is sensitive to noise and is prone to cause mode mixing [28,29,30,31]. Therefore, to solve the problems of EMD, many modified versions of EMD have been proposed, e.g., ensemble empirical mode decomposition [32], variational mode decomposition [33], and constrained mode decomposition [34]. These methods address some of the limitations of EMD, but they still struggle to handle multi-component signals with closely spaced modes. Then, Chen et al. [35] proposed a signal decomposition theorem based on the Hilbert transform, termed analytical mode decomposition (AMD). Both numerical simulations and laboratory experiments demonstrate that the AMD exhibits strong noise robustness and high accuracy in signal decomposition [36,37].

In this study, an improved HHT (IHHT) is proposed to identify the operational modal parameters of structures with closely spaced modes. First, the AMD is adopted to replace EMD for decomposing the measured response into several mono-component modes. The random decrement technique (RDT) [38] is then applied to the decomposed modes to obtain the free decay responses. Furthermore, the resulting free decay responses are analyzed by HT to identify the modal parameters of structures. Simulated data from a three-degree-of-freedom (3-DOF) system and a 24-story building, along with field test data from a cable-stayed bridge, are used to verify the practicality and reliability of the proposed method.

The remainder of this study is organized as follows. Section 2 provides a detailed review of the HHT and its limitations. Section 3 outlines the proposed IHHT method and describes the procedure for modal parameter identification. In Section 4, numerical case studies of a 3-DOF system and a high-rise building are presented to validate the accuracy and efficiency of the proposed method. Section 5 discusses the application of the proposed method for identifying the modal parameters of a large-span cable-stayed bridge with closely spaced modes.

## 2. Hilbert–Huang Transform

Hilbert–Huang transform (HHT) is a widely used signal processing method, particularly in fields such as civil, mechanical, and aerospace engineering. HHT is the combination of empirical mode decomposition (EMD) and Hilbert transform (HT). In this section, EMD and HT will be introduced separately.

### 2.1. EMD

The EMD algorithm is designed to decompose any nonlinear and non-stationary signals into several mono-component modes through a sifting process. These decomposed modes must meet two key conditions: (1) within the data range, the number of extrema and the number of zero crossings of a mono-component mode must either be equal or differ by no more than one; and (2) at any point, the envelopes defined by the local maxima and minima must be symmetric about the mean [20].

To decompose the measured response $f\left(t\right)$ into several mono-component modes, a sifting process is used in EMD, and its specific process is as follows. 

First, all local extrema of the input signal are identified, and the local maxima and minima are connected using cubic splines to generate the upper and lower envelopes. The average of these two envelopes is denoted as $m\left(t\right)$ and can be expressed as

(1) $$m\left(t\right)=\frac{f_{\max}\left(t\right)-f_{\min}\left(t\right)}{2}$$

where $f_{\max}\left(t\right)$ is the upper envelope, and $f_{\min}\left(t\right)$ is the lower envelope. 

Then, the difference between $m\left(t\right)$ and the input signal $f\left(t\right)$ can be calculated by

(2) $$c_{1}\left(t\right)=f\left(t\right)-m\left(t\right)$$

If $c_{1}\left(t\right)$ meets the two conditions mentioned above, it is considered the first extracted mono-component mode. If it does not meet the condition, the processes outlined in Equations (1) and (2) are repeated until $c_{1}\left(t\right)$ satisfies both conditions, and the extracted first mono-component mode is denoted as $x_{1}\left(t\right)$. After extracting $x_{1}\left(t\right)$ from the input signal, the remaining residual can be expressed as

(3) $$r_{1}\left(t\right)=f\left(t\right)-x_{1}\left(t\right)$$

where $r_{1}\left(t\right)$ is the remaining residual. 

Furthermore, $r_{1}\left(t\right)$ is used as the new input signal to repeat the above process until $x_{2}\left(t\right)$, $x_{3}\left(t\right)$, ⋯, and $x_{K}\left(t\right)$ are obtained. Therefore, the original signal $f\left(t\right)$ can be expressed as the sum of *K* decomposed mono-component modes, given by

(4) $$f\left(t\right)=\sum_{k=1}^{K}x_{k}\left(t\right)+r_{k}\left(t\right)$$

where $x_{k}\left(t\right)\;\left(k=1,2,\cdots ,K\right)$ is the *k*-th mono-component mode; and $r_{k}\left(t\right)$ is the final residue signal. The decomposed mono-component modes are arranged in order from high frequency to low frequency. More details about EMD can be found in Ref. [20].

### 2.2. Hilbert Transform

The Hilbert transform (HT) is a mathematical operation that transforms a real-valued signal into a complex-valued signal, enabling the extraction of phase and amplitude information from the signal. Given a real-valued signal $x_{k}\left(t\right)$, HT is defined as

(5) $${\hat{x}}_{k}(t)=\frac{P}{\pi }\int_{-\infty }^{\infty }\frac{x_{k}(\tau )}{t-\tau }d\tau$$

where ${\hat{x}}_{k}(t)$ represents the HT of the signal $x_{k}\left(t\right)$, *P* denotes the Cauchy principle value, and τ is the variable of integration. The integral operates as a convolution with the kernel $\frac{P}{\pi \left(t-\tau \right)}$, which acts to create a phase shift in the signal. Then, the analytic signal of $x_{k}\left(t\right)$ can be expressed as

(6) $$z(t)=x_{k}(t)+j{\hat{x}}_{k}(t)=A_{k}\left(t\right)e^{j\phi_{k}\left(t\right)}$$

with

(7) $$A_{k}(t)=\sqrt{{x_{k}}^{2}(t)+{{\hat{x}}_{k}}^{2}(t)}$$

(8) $$\phi_{k}(t)=\arctan\left({\hat{x}}_{k}(t)/x_{k}(t)\right)$$

in which $A_{k}(t)$ and ϕ(t) are the instantaneous amplitude and initial phase of $x_{k}\left(t\right)$. Therefore, the instantaneous frequency can be calculated by

(9) $$\omega (t)=\frac{1}{2\pi }\frac{d\phi (t)}{dt}$$

In HHT, EMD is sensitive to noise and sampling frequency and has limited capability in decomposing the measured response with close-spaced modes. These limitations result in the low signal decomposition accuracy of EMD, which in turn hinders the ability of HHT to identify the modal parameters of structures with closely spaced modes.

## 3. Improved HHT for Modal Parameter Identification 

To address the limitations of HHT, an improved HHT (IHHT) is proposed in this section and is used to identify the operational modal parameters of structures with closely spaced modes. The proposed IHHT consists of three main ingredients: analytical mode decomposition (AMD), random decrement technique (RDT), and Hilbert transform (HT).

### 3.1. AMD

AMD is a high-precision signal decomposition method that is used in place of EMD to decompose the response of structures with closely spaced modes [35]. Its core principle is to accurately separate a measured response into two-time functions using a bisecting frequency. Through multiple steps of separation, a measured response with closely spaced modes can be decomposed into multiple mono-component modes. 

Typically, the measured response is a multi-component amplitude-modulated and frequency-modulated signal, i.e.,

(10) $$f\left(t\right)=\sum_{k=1}^{K}x_{k}\left(t\right)=\sum_{k=1}^{K}A_{k}\left(t\right)e^{j\phi_{k}\left(t\right)}$$

where $f\left(t\right)$ is the measured response, $x_{k}\left(t\right)$ represents the original *k*-th mode, and *K* is the number of modes.

Assuming that the Fourier transform values of the decomposed mono-component modes are equal to those of the original modes, each mode of the measured response can be extracted as follows:

(11) $$\begin{array}{l} x_{1}^{\left(d\right)}(t)=s_{1}(t)-s_{0}(t),\;\ldots \\ x_{k}^{\left(d\right)}(t)=s_{k}(t)-s_{k-1}(t),\;\ldots \\ x_{K}^{\left(d\right)}(t)=f(t)-s_{k-1}(t) \end{array}$$

(12) $$s_{k}(t)=\sin\left(\omega_{bk}t\right)H\left[x(t)\cos\left(\omega_{bk}t\right)\right]-\cos\left(\omega_{bk}t\right)H\left[x(t)\sin\left(\omega_{bk}t\right)\right]\;(k=1,2,\ldots ,K-1)$$

where $s_{0}(t)=0$, $x_{k}^{\left(d\right)}(t)$ is the decomposed *k*-th mode, $\omega_{bk}$ represents the bisecting frequency, and H represents the Hilbert transform operator.

By repeatedly applying Equations (11) and (12), the measured response can be decomposed as follows:

(13) $$f\left(t\right)=x_{1}^{d}\left(t\right)+x_{2}^{d}\left(t\right)+\cdots +x_{k}^{d}\left(t\right)+\cdots +x_{K-1}^{d}\left(t\right)+x_{K}^{d}\left(t\right)+\eta \left(t\right)$$

where $x_{1}^{d}\left(t\right)\cdots x_{K}^{d}\left(t\right)$ represents decomposed mono-components modes, and $\eta \left(t\right)$ is the residual signal.

### 3.2. RDT

The random decrement technique (RDT) is a simple and easily implemented method for the analysis of vibrations of structures subjected to ambient excitations [38]. It can construct a free decay response by calculating the ensemble average of preselected sample segments from the measured response. The basic concept of the free decay response is founded on the idea that the random response of a structure subjected to ambient excitations consists of two components: a deterministic part and a random part [39]. By averaging enough samples of the same random response, the random part of the response will average out, leaving the deterministic part of the response.

By setting an appropriate triggering condition, the free decay response can be obtained by extracting segments of the decomposed *k*-th mode $x_{k}\left(t\right)$, i.e.,

(14) $$h\left(t\right)=\frac{1}{N}\sum_{i=1}^{n}x_{k}\left(t_{i}+s\right)$$

where $u\left(t\right)$ represents the free decay response, *N* is the total number of trigger points, and *s* is the time variable. Alternatively, zero up-crossings or down-crossings can be used as triggering conditions.

To effectively select the trigger level for RDT and enhance its accuracy with limited data samples, the following two processing methods are employed:

(1) Both zero up-crossings and down-crossings serve as triggering conditions, effectively doubling the number of segments included in the averaging process.
(2) The sampling frequency of the measured response is artificially increased using a curve fitting method to enhance the accuracy of detecting the triggering value.

### 3.3. HT for Modal Parameter Identification

After determining the free decay response for each mono-component mode using RDT, the Hilbert transform (HT) is applied to each free decay response $h\left(t\right)$ to identify the corresponding modal frequency $\omega_{n}$ and damping ratio ξ. $h\left(t\right)$ can be expressed as

(15) $$h\left(t\right)=Ae^{-\xi \omega_{n}t}\cos\left(\omega_{d}t+\phi_{0}\right)$$

where *A* is the amplitude, $\omega_{n}$ is the undamped natural frequency, $\omega_{d}$ is the damped angular frequency ($\omega_{d}=\omega_{n}\sqrt{1-\xi^{2}}$), $\phi_{0}$ is the initial phase lag, and ξ is the damping ratio. The analytical signal of the free decay response $h\left(t\right)$ can be expressed as [40]

(16) $$H_{a}\left(t\right)=h\left(t\right)+jH\left[h\left(t\right)\right]$$

where $j=\sqrt{-1}$, and $H\left[h\left(t\right)\right]$ is the HT of $h\left(t\right)$. The amplitude function $\left|H_{a}\left(t\right)\right|$ and the phase function $∠\left[H_{a}\left(t\right)\right]$ of the complex analytic signal $H_{a}\left(t\right)$ can be expressed as

(17) $$\left|H_{a}\left(t\right)\right|≅Ae^{-\xi \omega_{n}t}$$

(18) $$∠\left[H_{a}\left(t\right)\right]≅\omega_{d}t+\phi_{0}$$

By taking the derivative operation to the logarithm of the amplitude function and the phase function, Equations (17) and (18) are transformed into Equations (19) and (20), respectively, as follows:

(19) $$\ln\left|H_{a}\left(t\right)\right|=-\xi \omega_{n}t+\ln A\Rightarrow \frac{d\ln\left|H_{a}\left(t\right)\right|}{dt}=-\xi \omega_{n}$$

(20) $$\frac{d∠\left[H_{a}\left(t\right)\right]}{dt}=\omega_{d}=\omega_{n}\sqrt{1-\xi^{2}}$$

Therefore, the undamped natural frequency $\omega_{n}$ and the damping ratio ξ can be estimated according to the slope of the logarithm amplitude curve and the phase curve by using the linear least-square fit procedure, i.e.,

(21) $$\omega_{n}=\sqrt{{\left(\frac{d\ln\left|H_{a}\left(t\right)\right|}{dt}\right)}^{2}+{\left(\frac{d∠\left[H_{a}\left(t\right)\right]}{dt}\right)}^{2}}$$

(22) $$\xi =-\left(\frac{d\ln\left|H_{a}\left(t\right)\right|}{dt}\right)/\omega_{n}$$

However, it should be noted that the above HT-based modal parameter identification method is only applicable to single-degree-of-freedom systems with small damping, as their responses are mono-components with slowly varying amplitudes compared to the phase variations. 

### 3.4. Procedure of the Proposed Method for Modal Identification

**Figure 1:**
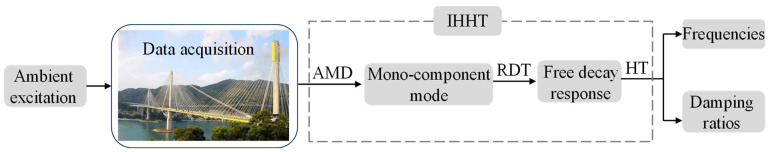
Flowchart of IHHT-based modal identification of structures with closely spaced modes.

As mentioned above, the proposed IHHT successfully overcame the limitations of the original HHT and is capable of identifying the operational modal parameters of structures with closely spaced modes. The procedure of identifying modal parameters using the IHHT is illustrated in Figure 1, and the specific implementation steps are summarized as follows:

(1) Input the measured response and determine the bisecting frequency of AMD based on the Fourier spectrum.
(2) Use AMD to decompose the measured response into several mono-component modes.
(3) Employ RDT to obtain the free decay response of each mono-component mode.
(4) Apply HT to the free decay responses to identify the frequencies and damping ratios of structures with closely spaced modes.

## 4. Numerical Examples

To demonstrate the accuracy and efficiency of the proposed IHHT-based operational modal identification method, a simulated three-degree-of-freedom (DOF) spring-mass system and a 24-story building subjected to white noise excitations are used for testing. The original HHT and SSI are two commonly used operational modal analysis methods, so they are adopted for comparison with the proposed method.

### 4.1. A 3-DOF Spring-Mass System

**Figure 2:**
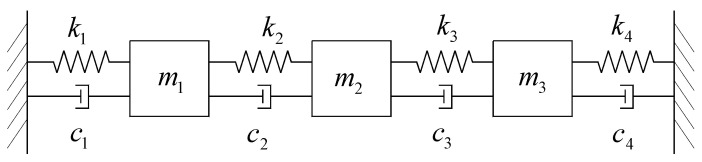
The simulated 3-DOF spring-mass system.

**Figure 3:**
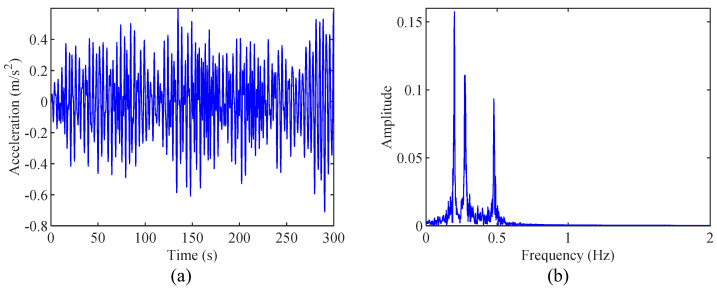
(**a**) The recorded acceleration response and (**b**) its corresponding spectrum.

The simulated 3-DOF spring-mass system is shown in Figure 2. The physical parameters of this system are $m_{1}=m_{3}=20000\;\mathrm{kg}$, $m_{2}=8000\;\mathrm{kg}$, $k_{1}=k_{3}=k_{4}=40\;\mathrm{kN}/m$, $k_{2}=15\;\mathrm{kN}/m$, $c_{1}=c_{4}=120\;N\cdot s/m$, and $c_{2}=c_{3}=90\;N\cdot s/m$. The theoretical modal frequencies and modal damping ratios are $f_{1}=0.1994\;\mathrm{Hz}$, $f_{2}=0.2759\;\mathrm{Hz}$, $f_{3}=0.4793\;\mathrm{Hz}$, $\xi_{1}=0.19\%$, $\xi_{2}=0.34\%$, and $\xi_{3}=0.45\%$. White noise excitation is applied to this 3-DOF spring-mass system, and the acceleration response of $m_{3}$ is recorded at a sampling frequency of 100 Hz. The recorded acceleration response of $m_{3}$ and its corresponding Fourier spectrum are shown in Figure 3a,b, respectively. As shown in Figure 3b, the first and second modes are close to each other.

**Figure 4:**
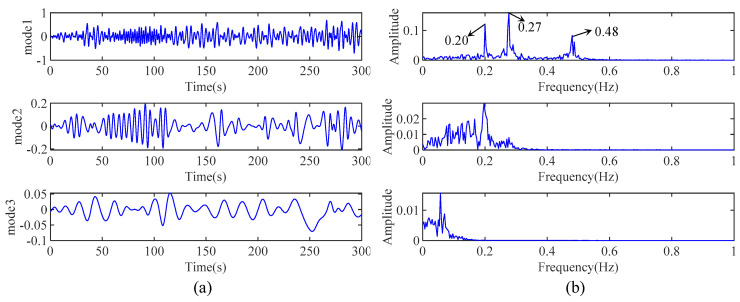
(**a**) The decomposed modes by EMD and (**b**) their Fourier spectrum.

Figure 4a,b represent the decomposed modes by EMD and their corresponding Fourier spectrum. As shown clearly in Figure 4b, the frequencies 0.20 Hz, 0.27 Hz, and 0.48 Hz are all coupled within mode 1. Therefore, the EMD decomposition results exhibit significant mode mixing, failing to separate each mode of the measured response individually. This phenomenon is caused by the inherent limitations of EMD. The poor signal decomposition accuracy of EMD leads to poor modal parameter identification results in the original HHT.

**Figure 5:**
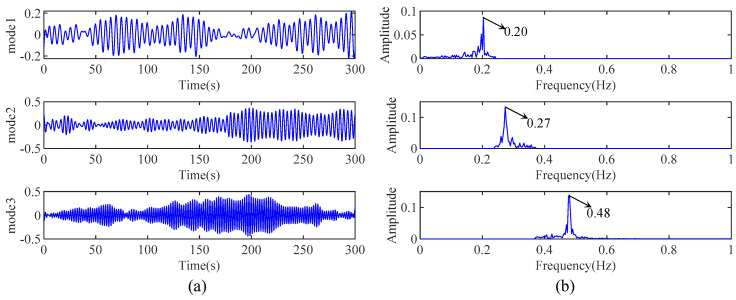
(**a**) The decomposed modes by AMD and (**b**) their Fourier spectrum.

Accordingly, Figure 5a,b represent the decomposed mono-component modes obtained from AMD and their corresponding Fourier spectrum, respectively. It can be observed that the AMD used in this study is capable of decomposing the measured response from the 3-DOF with closely spaced modes into mono-component modes. By comparing Figure 4 and Figure 5, it is evident that AMD can overcome the limitations of EMD. Therefore, the AMD in IHHT can be used to replace EMD to decompose the measured response with closely spaced modes into several mono-component modes.

**Figure 6:**
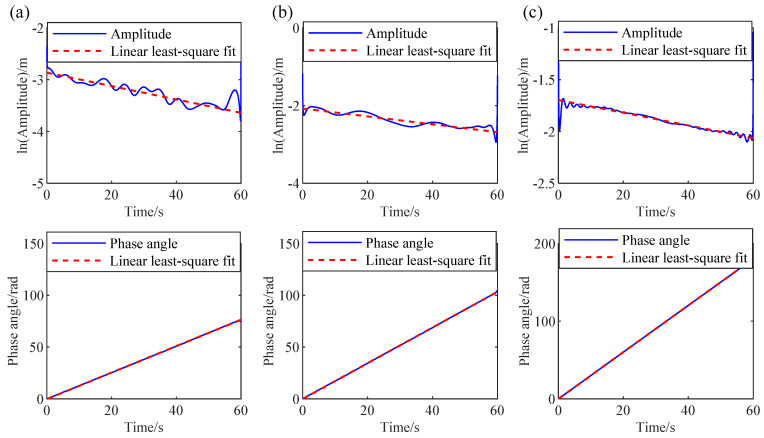
Logarithmic amplitude and phase curves in 3-DOF system: (**a**) mode 1, (**b**) mode 2, and (**c**) mode 3.

As mentioned above, AMD has accurately decomposed the measured response. Subsequently, RDT is applied to the decomposed mono-component modes to obtain the free decay response for each mode. Once the free decay responses are obtained by RDT, the natural frequencies and damping ratios of the 3-DOF system can be determined by the HT-based mono-component modal parameter identification method. The logarithmic amplitude and phase curves of the free decay response are shown in Figure 6, where the solid lines are the actual curves, and the dashed lines are the fitting curves by using the linear least-square fit procedure. Finally, the modal parameter identification results by the proposed IHHT method are listed in Table 1. As illustrated in Table 1, the natural frequencies and damping ratios identified by the proposed IHHT method are very close to the theoretical values. The identification error for the natural frequencies is less than 1%, and the error for the damping ratios is less than 6%.

Additionally, HHT and SSI are used to identify the modal parameters of the 3-DOF system, and their identification results are also listed in Table 1. It is clear that due to the low decomposition accuracy of EMD in HHT, mode mixing occurs, which results in HHT only identifying the first-order frequency and damping. The second and third-order modal parameters of the 3-DOF spring-mass system could not be identified. As a well-established operational modal identification method, SSI was able to identify the modal parameters of the simulated 3-DOF system with results that are also close to the theoretical values. The natural frequency identification error using SSI is less than 1%, and the damping ratio error is less than 11%.

Compared to the identification results of the original HHT and SSI methods, the proposed method achieves higher accuracy in modal parameter identification. Therefore, the proposed IHHT method is suitable for identifying the operational modal parameters of structures with closely spaced modes.

### 4.2. A High-Rise Building

**Figure 7:**
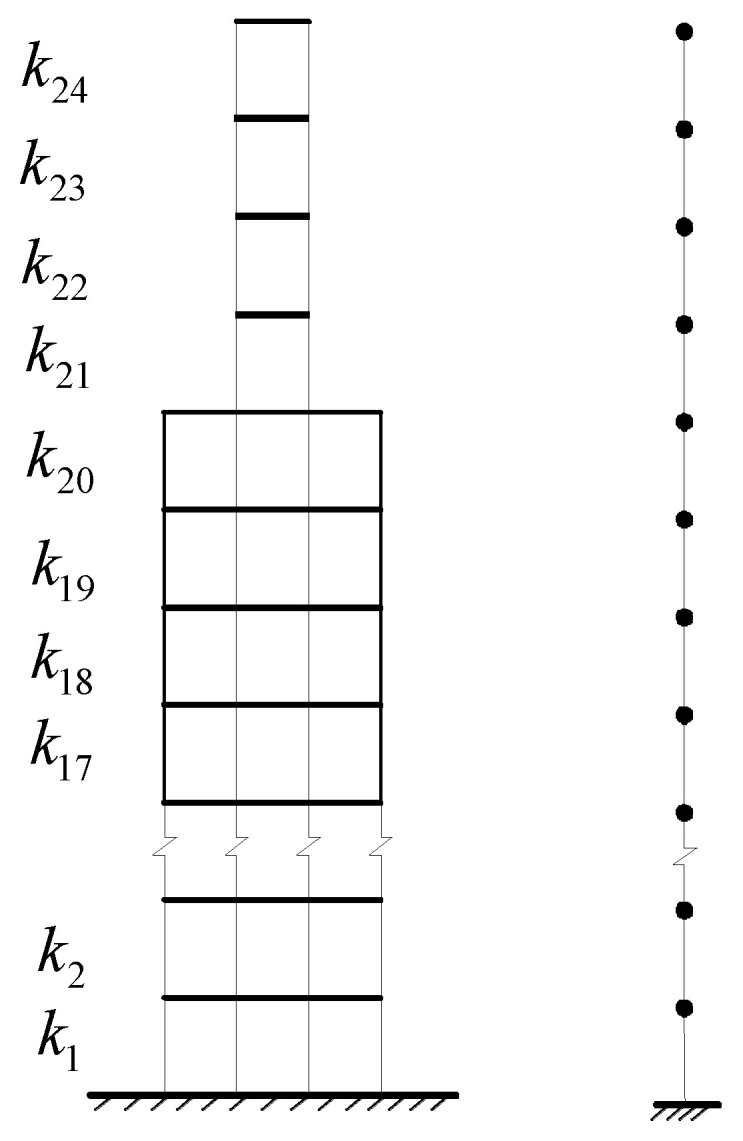
The simulated high-rise building with four additional lightweight appendages.

In order to further prove the effectiveness and accuracy of the proposed method in identifying the operational modal parameters of the civil engineering structures with closely spaced modes, simulation tests of a high-rise building are conducted in this section. As shown in Figure 7, the simulated building is a 20-story shear structure with a 4-story additional lightweight appendage on top. The physical parameters of the building are listed as follows:

The mass and stiffness from the 1st floor to the 20th floor are $m_{1}=m_{2}=\cdots =m_{20}=3\times 10^{6}\;\mathrm{kg}$ and $k_{1}=k_{2}=\cdots =k_{20}=10^{9}\;N/m$, respectively. The mass and stiffness of the four-story lightweight appendage are $m_{21}=m_{22}=m_{23}=m_{24}=3\times 10^{5}\;\mathrm{kg}$ and $k_{21}=k_{22}=k_{23}=k_{24}=4.85\times 10^{6}\;N/m$, respectively. The mass ratio and damping ratio between the lightweight appendage and the building floors are 0.1 and $4.85\times 10^{-3}$, respectively. The theoretical first four modal frequencies are $f_{1}=0.2021\;\mathrm{Hz}$, $f_{2}=0.2433\;\mathrm{Hz}$, $f_{3}=0.6313\;\mathrm{Hz}$, and $f_{4}=0.6766\;\mathrm{Hz}$. Due to the considerable difference in mass and stiffness between the lightweight appendage and the building floors, the appendage has a notable impact on the building’s dynamic characteristics, resulting in the close distribution of the first two natural frequencies. We assumed that the first four modal damping ratios are 1%, i.e., $\xi_{1}=\xi_{2}=\xi_{3}=\xi_{4}=1\%$, and the other modal damping ratios are

(23) $$\xi_{i}=\min\left\{\frac{f_{k}}{100f_{1}},\;0.1\right\},\;k=5,\;24$$

where $f_{k}$ is the *k*-th frequency. 

**Figure 8:**
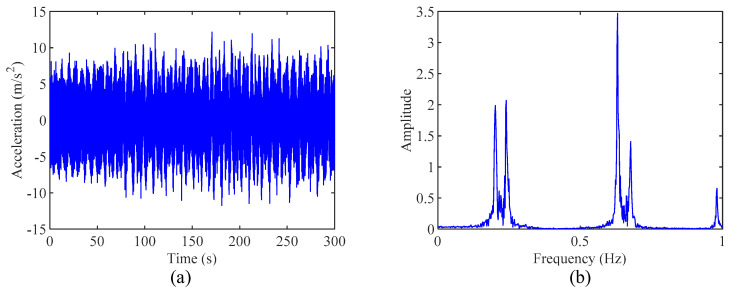
(**a**) The acceleration time history at the top of the lightweight appendage and (**b**) its corresponding Fourier spectrum.

Under the action of the white-noise ground ambient excitation with the frequency range of 0.0 Hz~50.0 Hz and the magnitude of 0.1 m/s^2^, the acceleration time history at the top of the lightweight appendage is calculated using the Wilson-θ method, as shown in Figure 8a. The corresponding Fourier spectrum is presented in Figure 8b. It is evident that the first and second modes of the simulated building are closely packed, as are the third and fourth modes.

**Figure 9:**
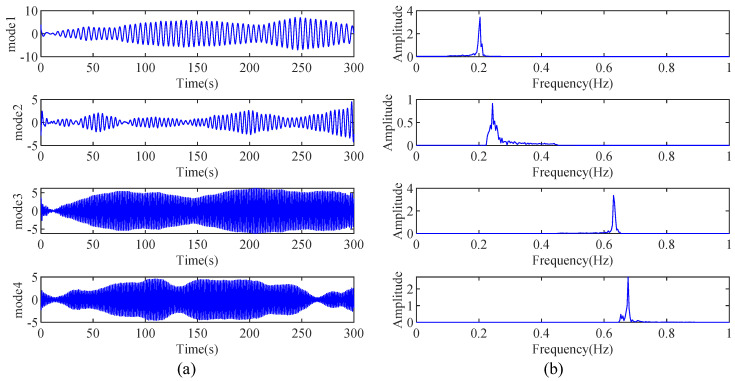
(**a**) The decomposed mono-component modes by AMD and (**b**) their Fourier spectrum.

**Figure 10:**
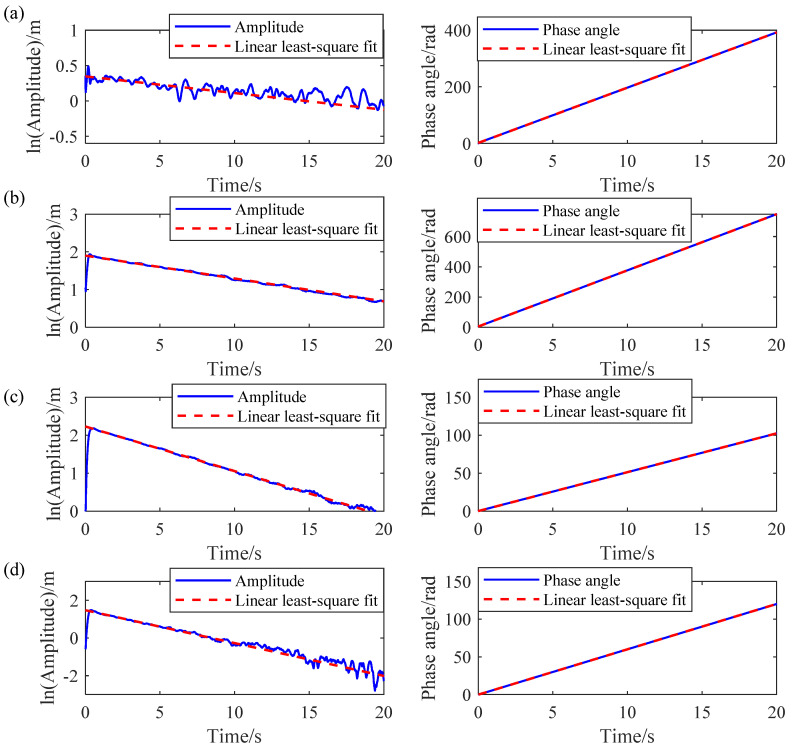
Logarithmic amplitude and phase curves in high-rise building: (**a**) mode 1, (**b**) mode 2, (**c**) mode 3, and (**d**) mode 4.

The proposed IHHT-based method is employed to identify the modal parameters of the simulated building. First, the AMD is used to decompose the measured acceleration time history into several mono-component modes. The decomposed modes and their corresponding Fourier spectra are shown in Figure 9a,b, respectively. It is evident that AMD can accurately decompose the measured response with closely spaced modes into distinct mono-component modes, without any mode mixing between them. Then, RDT is applied to the decomposed modes to obtain the free decay responses. Based on the obtained free decay responses, the modal parameters of the simulated high-rise building are identified by using the HT-based modal parameter identification method, and the results are shown in Table 2. The logarithmic amplitude curves and the phase curves of the free decay responses are shown in Figure 10a–d. In these figures, the dotted lines are the actual curves, and the solid lines are the fitting curves by using the least-square method. As shown in Table 2, the modal parameter identification results from the proposed method are almost identical to the theoretical values. The maximum identification errors of the proposed method for natural frequencies and damping ratios are 1% and 11%, respectively. Based on the study by Su et al. [41], the maximum allowable errors for natural frequencies and damping ratios are 2% and 20%, respectively. It is evident that the proposed method meets these requirements well.

Original HHT and SSI are also adopted to identify the modal parameters of this high-rise building. Due to mode mixing in the decomposed modes by HHT, it can only identify the first-order natural frequencies and damping ratios, with relatively poor accuracy. In addition, as shown in Table 2, SSI is capable of identifying the first four-order modal parameters of the simulated building, but its accuracy is not as high as that of the proposed method. There is no doubt that SSI is an effective method for operational modal parameters analysis, but it requires the manual presetting of two parameters, the order and block rows, which affects its identification accuracy.

## 5. Engineering Application in a Long-Span Cable-Stayed Bridge

**Figure 11:**
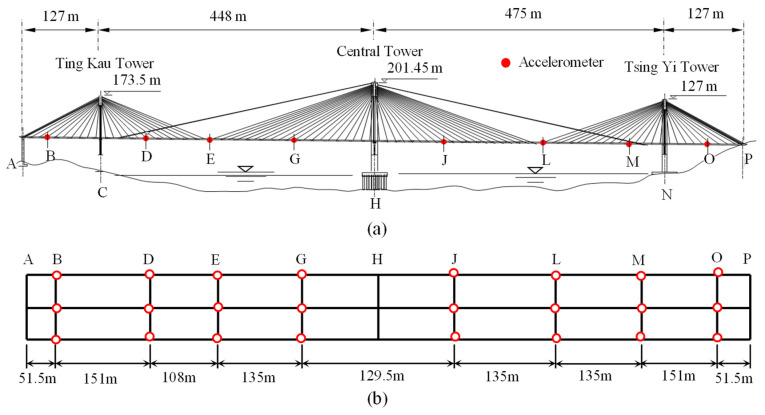
(**a**) Ting Kau Bridge and (**b**) layout of accelerometers installed on bridge deck.

To further validate the practicality of the proposed method for identifying the modal parameters of civil engineering structures with closely spaced modes, the Ting Kau Bridge (TKB), a long-span cable-stayed bridge, is selected as a case study. As shown in Figure 11a, TKB is a three-tower, four-span cable-stayed bridge. The main spans of this bridge measure 448 m and 475 m, while the two side spans are each 127 m in length. The three slender, single-leg towers stand at heights of 170 m, 194 m, and 158 m, respectively. The upper part of the bridge deck is a concrete slab, while the underside features two longitudinal steel girders along the edges of the deck, with steel cross girders spaced at 4.5 m intervals. The bridge deck is divided into two lanes, each 18.8 m wide, and it is supported by 384 stay cables arranged in four cable planes. In addition, over 230 sensors were permanently installed on the bridge after its completion in 1999. These sensors include accelerometers, displacement transducers, anemometers, strain gauges, weigh-in-motion sensors, temperature sensors, and GPS. Figure 11b shows the arrangement of 24 uniaxial accelerometers across eight deck sections of the TKB. Accelerometers (2, 5, 8, 11, 14, 17, 20, 23) installed on the central cross-girder measure transverse acceleration, while the remaining accelerometers measure vertical acceleration. Since the proposed method can only process single-channel data, it is applied to each channel separately, and then the average of the modal frequencies and the damping ratios are calculated. In this study, data collected from 24 accelerometers installed on the bridge deck are utilized. The dataset, recorded during Typhoon York with a wind speed of 15.91 m/s, lasts one hour, and the sampling frequency is 25.6 Hz.

**Figure 12:**
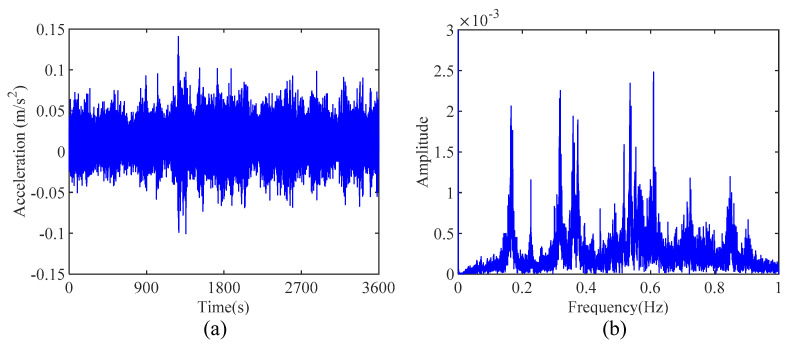
(**a**) The measured acceleration time histories by accelerometer 17 and (**b**) its corresponding Fourier spectrum.

**Figure 13:**
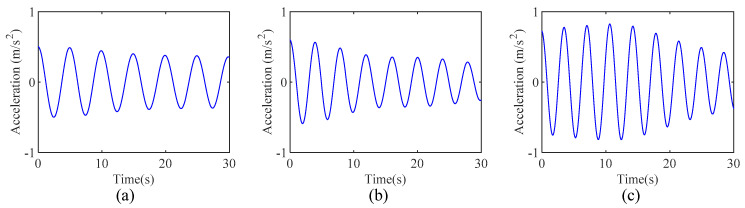
The obtained free decay response by RDT: (**a**) mode 1, (**b**) mode 2, and (**c**) mode 3.

**Figure 14:**
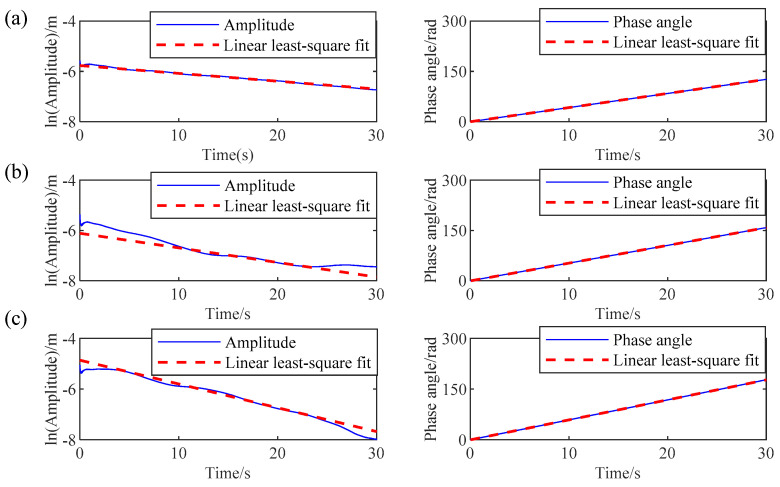
The logarithmic amplitude and phase curves using the data from sensor 10: (**a**) mode 1, (**b**) mode 2, and (**c**) mode 3.

The response measured by accelerometer 17 and its corresponding Fourier spectrum are illustrated in Figure 12a,b, respectively. Due to the large span of this bridge, its frequency modes are closely spaced, as shown in Figure 12b. The proposed IHHT-based method is employed to identify the modal parameters of TKB. First, the measured acceleration response is decomposed into several mono-component modes. In this study, only the first three frequencies are considered. Therefore, it is sufficient to use AMD to extract the first three modes from the measured response. Following the proposed method, RDT is then applied to the decomposed modes to extract the free decay response of each mode, and the results are shown in Figure 13. Furthermore, HT is applied to the obtained free decay responses to calculate the instantaneous phase angle and logarithmic amplitude of each mode. Figure 14 displays the calculated instantaneous phase angle and logarithmic amplitude, along with the corresponding fit lines obtained using the linear least-squares fitting technique. The modal damping ratios and natural frequencies can subsequently be identified from the slopes of these two fit lines. Table 3 summarizes the modal parameter identification results by the proposed method and compares them with the finite element analysis results [42]. As shown in Table 3, the first three frequencies identified by using the proposed method are 0.166 Hz, 0.227 Hz, and 0.263 Hz, respectively, which is in good agreement with the identified results by the finite element analysis. This phenomenon indicates that the proposed method can be utilized to identify the natural frequencies and damping ratios of civil engineering structures with closely spaced modes.

## 6. Conclusions

To identify the operational modal parameters of structures with closed-spaced modes, this study proposed an accurate and efficient method based on IHHT. In this method, AMD is first used to replace EMD for decomposing the measured response into several mono-component modes. Then, the resulting mono-component modes are analyzed by the random decrement technique (RDT) to obtain the free decay responses. Finally, the HT is applied to the free decay response to identify the modal parameters of structures with closed-spaced modes under ambient excitations.

Numerical examples of a simple 3-DOF system and a 24-story building under white noise excitations are adopted to demonstrate the accuracy and efficiency of the proposed method. The results show that the proposed method is capable of identifying the modal parameters of structures with closely spaced modes. Additionally, the proposed method offers higher accuracy in modal parameter identification compared to existing methods. 

The field measurement response from the Ting Kau bridge under the typhoon excitations is used to demonstrate the practicality of the proposed method. The proposed method has been proven to be straightforward and effective, making it suitable for identifying the operational modal parameters of existing long-scale structures with closely spaced modes.

It should be noted that the cutoff frequency of AMD needs to be manually preset based on the Fourier spectrum. In a high-noise environment, the cutoff frequency may be inaccurately preset, which could affect the accuracy of the modal parameter identification.

## Figures and Tables

**Table 1:** The identified modal parameters of the 3-DOF system.

Methods	Identification Results of Mode 1/Relative Error		Identification Results of Mode 2/Relative Error		Identification Results of Mode 3/Relative Error	
	Natural Freq. (Hz)	Damping Ratio (%)	Natural Freq. (Hz)	Damping Ratio (%)	Natural Freq. (Hz)	Damping Ratio (%)
Theoretical values	0.199	0.19	0.275	0.34	0.479	0.45
Proposed IHHT	0.198/0.5%	0.18/5.2%	0.276/0.4%	0.32/5.8%	0.477/0.4%	0.44/2.2%
HHT	0.214/7.5%	0.25/31.5%	____	____	____	____
SSI	0.198/0.5%	0.21/10.5%	0.277/0.7%	0.32/5.8%	0.476/0.6%	0.47/4.4%

**Table 2:** The identified modal parameters in the high-rise building case.

Mode Number	Theoretical Values		Proposed Method/Relative Error		HHT/Relative Error		SSI/Relative Error	
	Natural Freq.	Damping Ratio (%)	Natural Freq.	Damping Ratio (%)	Natural Freq.	Damping Ratio (%)	Natural Freq.	Damping Ratio (%)
Mode 1	0.202	1.00	0.204/0.9%	1.08/8%	0.187/7.4%	1.9/90%	0.201/0.5%	0.91/9%
Mode 2	0.243	1.00	0.244/0.4%	0.91/9%	____	____	0.247/1.6%	1.24/24%
Mode 3	0.631	1.00	0.629/0.3%	0.89/11%	____	____	0.634/0.5%	1.15/15%
Mode 4	0.677	1.00	0.680/0.4%	0.93/7%	____	____	0.673/1.1%	0.82/18%

**Table 3:** The modal parameter identification results of TKB.

Mode No.	Finite Element Calculation	Proposed Method	
	Natural Freq. (Hz)	Natural Freq. (Hz)	Damping Ratio (%)
1	0.170	0.166	2.27
2	0.226	0.227	1.76
3	0.262	0.263	2.06

## Data Availability

Data are contained within the article.
